# Enhanced Inhibition of Prostate Tumor Growth by Dual Targeting the Androgen Receptor and the Regulatory Subunit Type Iα of Protein Kinase A *in Vivo*

**DOI:** 10.3390/ijms140611942

**Published:** 2013-06-04

**Authors:** Iris E. Eder, Martina Egger, Hannes Neuwirt, Christof Seifarth, Danilo Maddalo, Andreas Desiniotis, Georg Schäfer, Martin Puhr, Jasmin Bektic, Andrew C. B. Cato, Helmut Klocker

**Affiliations:** 1 Division of Experimental Urology, Innsbruck Medical University, 6020 Innsbruck, Anichstraße 35, Austria; E-Mails: martina.egger@gmail.com (M.E.); christof.seifarth@i-med.ac.at (C.S.); a_desiniotis@hotmail.com (A.D.); georg.schaefer@i-med.ac.at (G.S.); martin.puhr@i-med.ac.at (M.P.); jasmin.bektic@uki.at (J.B.); helmut.klocker@uki.at (H.K.); 2 Department of Internal Medicine IV—Nephrology and Hypertension, Innsbruck Medical University, 6020 Innsbruck, Anichstraße 35, Austria; E-Mail: hannes.neuwirt@i-med.ac.at; 3 Oncotyrol Center for Personalized Cancer Medicine GmbH, Karl-Kapferer-Straße 5, 6020 Innsbruck, Austria; 4 Karlsruhe Institute of Technology, Institute of Toxicology and Genetics, Toxicology and Genetics, Hermann-von-Helmholtz-Platz 1, 76344 Eggenstein-Leopoldshafen, Germany; E-Mails: danilo.maddalo@kit.edu (D.M.); andrew.cato@kit.edu (A.C.B.C.)

**Keywords:** androgen receptor, cAMP-dependent protein kinase A, regulatory subunit type Iα, prostate cancer, antisense molecules, dual targeting, LNCaP xenografts, castration resistance

## Abstract

Progression to castration resistance is a major problem in the treatment of advanced prostate cancer and is likely to be driven by activation of several molecular pathways, including androgen receptor (AR) and cyclic AMP-dependent protein kinase A (PKA). In this study, we examined the therapeutic efficacy of a combined inhibition of the AR and the regulatory subunit type Iα (RIα) of protein kinase A with second generation antisense oligonucleotides (ODNs) in androgen-sensitive LNCaP and castration-resistant LNCaPabl tumors *in vivo*. We found that targeting the AR alone inhibited LNCaP, as well as LNCaPabl tumors. Combined inhibition resulted in an improved response over single targeting and even a complete tumor remission in LNCaPabl. Western blot analysis revealed that both ODNs were effective in reducing their target proteins when administered alone or in combination. In addition, treatment with the ODNs was associated with an induction of apoptosis. Our data suggest that dual targeting of the AR and PKARIα is more effective in inhibiting LNCaP and LNCaPabl tumor growth than single treatment and may give a treatment benefit, especially in castration-resistant prostate cancers.

## 1. Introduction

Androgen receptor (AR) activation classically acts through binding of androgens, which regulate normal prostate development and function. In prostate cancer, the AR plays an essential role in driving tumor progression, even under hormone ablated conditions. So far, there are five fundamental and non-mutually exclusive mechanisms known, which are thought to mediate aberrant AR activation in castration-resistant prostate cancer (CRPC) (recently reviewed by [1] and [2]). These include low levels of androgens remaining in the tumor tissue despite hormone ablation, which may in part result from *in situ* steroidogenesis [3], mutations in the AR gene, broadening the ligand binding spectrum of the receptor [4–6], AR overexpression [7], altered expression of co-regulatory molecules [8] and, finally, crosstalk with other intracellular signaling pathways [9–11]. This leads to a major problem in the treatment of advanced prostate cancer, where hormone ablation is one of the most effective and most commonly used therapies, since the majority of tumors eventually relapse and progress to a castration-resistant stage [12–14]. Hence, with regard to therapy, one major question is how aberrant AR activation can be effectively prevented in prostate cancer cells. Several preclinical studies have revealed that inhibiting AR expression by itself through small antisense molecules is effective in inhibiting prostate tumor growth [15–22]. In addition, a panel of novel drugs has been developed, which aim at directly targeting the AR or intervening with androgen synthesis [23]. A recent review on the outcome of phase III clinical trials, in fact, confirms that targeting the AR can improve survival of patients with metastatic CRPC [24].

Recent findings by Lee and coworkers revealed that inhibition of AR activation or AR knockdown results in an unwanted accumulation of AR-negative stem/progenitor cells, which do not only escape AR targeting therapy, but are even stimulated by it [25]. These data suggest that solely targeting the AR may not be sufficient for effective treatment of prostate cancer. In a previous study, we investigated the short-term effects of dual targeting of the AR with the regulatory subunit type I alpha (RIα) of protein kinase A (PKA) in androgen sensitive (LNCaP) and castration-resistant (LNCaPabl) prostate cancer cell lines *in vitro* [21]. In particular, we showed that inhibition of AR expression with small interference RNA molecules (siRNAs) was effective in inhibiting LNCaP and LNCaPabl cells and that this anti-proliferative effect could be further enhanced by simultaneous targeting of PKARIα. Moreover, AR and PKARIα were found to be co-expressed and co-activated in human prostate cancer tissue, suggesting that dual targeting of these two molecules is preferable to single treatment.

PKA is a heterotetrameric protein consisting of two major isoforms, PKA-I and PKA-II, which have different regulatory subunits, termed RI and RII. Each regulatory subunit, in turn, has four different subunit genes (RIα, RIβ, RIIα and RIIβ), which determine tissue distribution and biochemical properties of the respective PKAs. PKARIα is the regulatory subunit of PKA-I, and its over expression is associated with poor prognosis in prostate cancer [26,27]. There is evidence that the two isoforms exert distinct functions in regulating cell growth and differentiation. PKA-I is mainly overexpressed in cancer cells, whereas PKA-II is preferentially found in differentiated tissues [28]. Correspondingly, downregulation of PKARIα with the antisense oligonucleotide, named GEM231, induced cell growth arrest, apoptosis and differentiation *in vitro* and *in vivo*. In addition, GEM231 has also been investigated for its utility in combination therapies with common cytotoxic drugs [28].

Moreover, PKA was found to cross-talk with the AR, suggesting that it may have an impact on CRPC [29]. Several studies have shown that the AR can be activated in the presence and absence of androgens by cyclic AMP (cAMP)-dependent PKA [10,30–32]. Nazareth and Weigel, for instance, have observed that PKA activation by forskolin can activate the AR in an androgen-independent manner [10]. In addition, Sadar and coworkers have shown that forskolin increases PSA expression in the presence of a functional AR [31]. In our previous *in vitro* study, we found out that downregulation of the AR resulted in reduced protein levels of PKARIα and diminished PKA activity, and *vice versa*, silencing PKARIα reduced AR and PSA, suggesting that the two pathways are likely interacting with each other. In addition, we found out that inhibition of the expression of PKARIα abolished the agonistic effect of the antiandrogen bicalutamide in LNCaPabl cells [21]. Dual targeting of AR and PKA signaling pathways, therefore, seems to be a reliable treatment approach for CRPC.

The objective of the present study was to explore the therapeutic efficacy of combination treatment with second generation oligodeoxynucleotides (ODNs) targeting the AR and PKARIα in androgen-sensitive LNCaP and castration-resistant LNCaPabl tumors *in vivo.* We show that combined treatment with ODN_AR and ODN_PKA results in significantly higher growth inhibition of LNCaP and LNCaPabl xenograft tumors, compared to single treatments. Moreover, our results revealed that dual targeting is especially effective in LNCaPabl tumors, where combination treatment resulted in complete tumor remission.

## 2. Results and Discussion

### 2.1. Single or Dual Targeting of AR and PKARIα with Second Generation ODNs Inhibits Prostate Cancer Cell Growth *in Vitro* by Induction of Apoptosis

Previous *in vitro* experiments by our group [21] have shown an enhanced effect of combined targeting of AR and PKARIα over single treatments using small interference RNAs (siRNAs). Due to several limitations and uncertainties in the *in vivo* use of siRNAs [33], we decided in favor of mixed backbone ODNs to investigate the *in vivo* effect of this dual targeting approach. Second generation ODNs have already reached acceptable safety and efficacy standards in several nonclinical and clinical studies [34].

**Figure 1:**
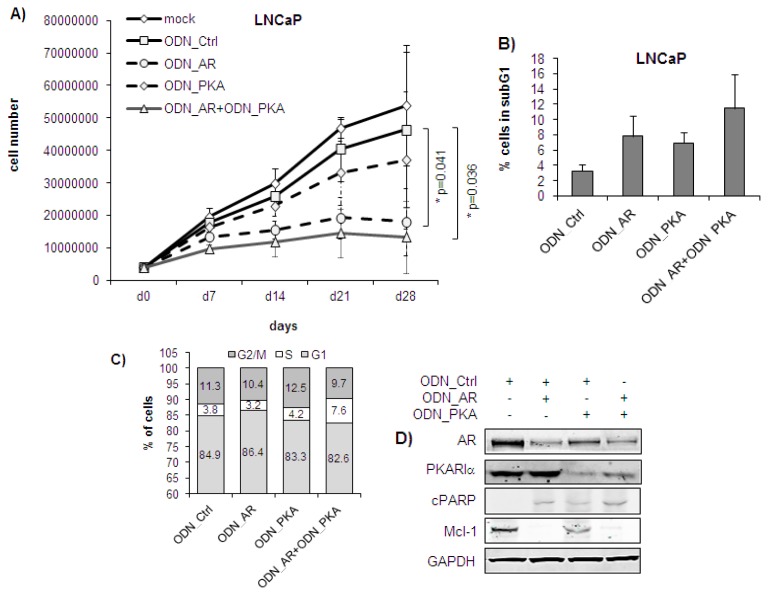
Effects of ODN_AR and ODN_PKA alone and in combination in LNCaP cells *in vitro*. Dual targeting of LNCaP cells was performed by transfection with ODN_AR + ODN_PKA (250 nM + 250 nM) over four weeks using oligofectamine (mock). Effects were compared with those of a non-targeting control oligodeoxynucleotide (ODN) (500 nM ODN_Ctrl). To estimate the effects of single targeting, ODN_AR or ODN_PKA were administered at a concentration of 250 nM each together with 250 nM of the ODN_Ctrl, respectively. Transfections were performed three times every other day in the first week and two times in weeks 2–4. After four weeks of treatment, cells were harvested for flow cytometry and Western blotting. (**A**) LNCaP cells treated with the ODN_AR alone or in combination with ODN_PKA were significantly inhibited compared to the mock control. Cells were counted weekly with a Casy cell counter after harvesting with trypsin, and the cell number was displayed cumulatively. *p < 0.05, two-way ANOVA; (**B**) The percentage of LNCaP cells in the subG1 phase was quantified by Nicoletti staining and subsequent flow cytometric measurement after four weeks of treatment; (**C**) Cell cycle distribution was measured after treatment of cells over four weeks. Cells were stained with propidium iodide and analyzed by flow cytometry. The graph shows mean percentages of cells in G1, S and G2M phases out of three separate experiments; (**D**) Expression of androgen receptor (AR), PKARIα, cPARP and Mcl-1 was determined by Western blotting and normalized to expression levels of glyceraldehyde 3-phosphate dehydrogenase (GAPDH). Representative images were taken out of three independent experiments.

Before testing the ODNs *in vivo*, we evaluated their effects in cultured LNCaP and LNCaPabl cells *in vitro*. To simulate conditions to be used *in vivo*, a long-term experiment was performed over four weeks. Cells were transfected with the ODNs either alone or in combination three times in the first week and two times in the following three weeks. Each time when control cells became confluent, cells of all treatment groups were trypsinized simultaneously and replated at an equal cell density. Effects on cell growth, target protein expression and apoptosis were compared with those obtained with an unspecific control (ODN_Ctrl). As illustrated in Figure 1A, ODN_AR alone significantly decreased the number of LNCaP cells to 33.6% of the control after four weeks of treatment (* *p* = 0.041, two-way ANOVA). ODN_PKA alone was less effective than ODN_AR, resulting in a decrease of cell number to 68.8% of the control at day 28 (*p* < 0.05). However, combined treatment of LNCaP cells with ODN_AR and ODN_PKA over four weeks resulted in a statistically significant cell number reduction (day 28: 24.6% of control, * *p* = 0.036, two-way ANOVA). Note that this inhibition was only slightly stronger than ODN_AR treatment alone, suggesting that AR knockdown alone is highly efficient in LNCaP cells. Corresponding to the effect on cell counts, ODN_AR alone increased the number of apoptotic cells 2.4-fold compared to control (*p* > 0.05); the ODN_PKA alone induced a 2.1-fold increase (*p* > 0.05). Combined treatment of LNCaP cells with ODN_AR and ODN_PKA over four weeks even potentiated the effect, yielding a 3.5-fold increase in the number of apoptotic cells compared to the control (*p* > 0.05, Figure 1B). However, the difference between single and dual targeting was not statistically significant. Analysis of cell cycle distribution revealed that the percentages of cells in the G1 phase were not substantially changed after treatment (Figure 1C). However, there was a statistically significant increase of cells in the S phase in the combined treatment group (7.6%) compared to the ODN_Ctrl (3.8%, *p* = 0.037), which was accompanied by a significant decrease of cells in the G2/M phase (ODN_AR + ODN_PKA: 9.7% compared to the ODN_Ctrl: 11.3%, *p* = 0.001), indicating a deterioration of S to G2/M transition by dual targeting. Most notably, this increase in the S phase was not observed in cells after single targeting with ODN_AR (3.2%) or ODN_PKA (4.2%), respectively. Determination of target protein expression by Western blotting revealed efficiently reduced AR protein levels after treatment with ODN_AR alone (29.6% of the ODN_Ctrl) or the combination of ODN_AR and ODN_PKA (19.8% of the ODN_Ctrl), respectively (Figure 1D). Corresponding with the effects seen on cell numbers, there was no significant difference in AR protein expression between single and combined targeting. Similarly, PKARIα protein levels were reduced after single use of ODN_PKA (31.1% of the control), as well as after combined use of ODN_PKA and ODN_AR (28.5% of the control). Interestingly, treatment with ODN_PKA also resulted in a decrease of AR protein levels (39.7% compared to ODN_Ctrl), confirming our previous *in vitro* findings that AR and PKARIα are likely interacting with each other [21]. In addition, we found that the apoptosis marker, cPARP, was increased, whereas the anti-apoptotic Mcl-1 protein was decreased in LNCaP cells when treated with the ODNs alone or in combination. Although we could not see any differences in cPARP or Mcl-1 expression levels between single and dual targeting, these data confirmed induction of apoptosis by single or combined targeting of the AR and PKARIα, respectively. Overall, the ODNs against AR and PKARIα had similar effects in LNCaP cells as those previously described for siRNAs with regard to the inhibition of target protein expression, reduced cell numbers and induction of apoptosis.

**Figure 2:**
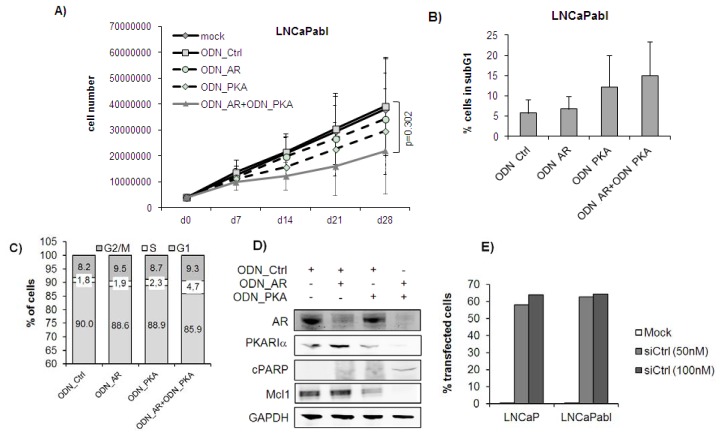
Single and dual targeting of AR and PKARIα in LNCaPabl *in vitro*. The treatment protocol described for LNCaP cells in the legend to Figure 1 was applied to LNCaPabl. (**A**) Cell number was counted weekly with a Casy cell counter after harvesting the cells with trypsin and displayed cumulatively; (**B**) The percentage of LNCaP cells in the subG1 phase was quantified by Nicoletti staining and subsequent flow cytometric measurement at the end of treatment (day 28); (**C**) Cell cycle distribution was measured after treatment of cells over four weeks. Cells were stained with propidium iodide and analyzed by flow cytometry. Graph shows mean percentages of cells in G1, S and G2M phases out of three separate experiments; (**D**) Expression of AR, PKARIα, cPARP and Mcl-1 was determined by Western blotting and normalized to expression levels of glyceraldehyde 3-phosphate dehydrogenase (GAPDH) after four weeks of treatment. Representative images were taken out of two independent experiments; (**E**) Transfection efficiency was measured by flow cytometry after transfection of cells with a fluorescently-labeled single-stranded control RNA-ODN (siCtrl) over 24 h.

In LNCaPabl cells, the growth-inhibitory effects of the ODNs either alone or in combination were generally weaker than in LNCaP cells. As depicted in Figure 2A, single targeting with ODN_AR or ODN_PKA yielded a moderate reduction of cell number to 83.3% (*p* > 0.05) and 72.0% (*p* > 0.05) of control at day 28, respectively. Combined treatment with ODN_AR and ODN_PKA reduced the cell number to 53.5% compared to the control at day 28. However, this was not statistically significant (*p* = 0.302, two-way ANOVA). Consistent with the effect on cell growth and fitting to the hormone ablation-resistant phenotype of LNCaPabl cells, ODN_AR alone had a minor effect on apoptosis in these cells (1.16-fold change *versus* control, *p* > 0.05). This finding is in line with previous data on increased resistance of LNCaPabl tumor cells to induction of apoptosis [35]. ODN_PKA treatment, on the other hand, showed a trend towards increased number of apoptotic cells (2.1-fold change *versus* control, *p* > 0.05), which was slightly enhanced by combined treatment with ODN_AR and ODN_PKA (2.6-fold change *versus* control). However, these changes did not reach statistical significance (*p* > 0.05, Figure 2B). Similarly, the strongest effects on cPARP and Mcl-1 levels were seen in cells treated with the combined treatment regimen, whereas there were only minor expression changes in cells treated with the ODN_AR alone, as revealed by Western blotting (Figure 2D). Cell cycle analysis revealed a significant increase of cells in the S phase in the combined treatment group (4.7%) compared to the ODN_Ctrl (1.8%, *p* = 0.037), which was accompanied by a (statistically not significant) decrease of cells in the G1 phase (Figure 2C). In addition, we found that target protein expression was effectively inhibited by the ODNs. Both the ODN_AR, as well as the ODN_PKA almost totally reduced AR and PKARIα protein levels, respectively (Figure 2D), suggesting that the moderate effects on cell numbers and apoptosis are unlikely due to inefficient transfection of LNCaPabl cells. To further strengthen this finding, we determined transfection efficiencies of LNCaP and LNCaPabl cells using a fluorescently-labeled single-stranded control RNA-ODN. As shown in Figure 2E, we found that about 60% of cells were transfected with even slightly higher transfection efficiency in LNCaPabl compared with LNCaP cells.

### 2.2. Combined Targeting of AR and PKARIα Results in Improved Growth Inhibition over Single Targeting in Androgen Sensitive LNCaP Tumors *in Vivo*

**Figure 3:**
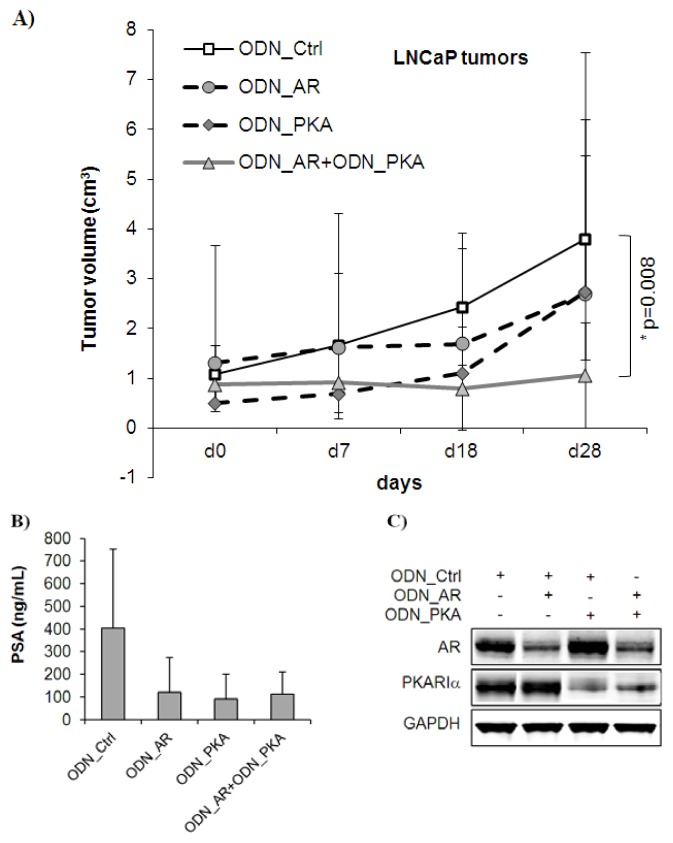
Single and combined targeting of AR and PKARIα in LNCaP tumors *in vivo*. Tumors were established by subcutaneous injection of LNCaP tumor cells and matrigel in a ratio of 1:1. ODNs were injected intraperitoneally at a final concentration of 10 mg/kg mouse three times in the first and two times in the following three weeks. For single treatments, ODNs were administered at a concentration of 5 mg/kg mouse together with 5 mg/kg mouse ODN_Ctrl. In the control group, mice received 10 mg/kg mouse ODN_Ctrl. (**A**) Tumor volumes were measured with a caliper weekly. The graph shows mean tumor volume ± S.E.M. After four weeks, tumors, which were treated with the combination of ODN_AR and ODN_PKA, were significantly smaller (*n* = 12, * *p* = 0.008, Mann Whitney-*U* test) than those treated with the ODN_Ctrl (*n* = 8). Single treatment with either the ODN_AR (*n* = 8) or the ODN_PKA (*n* = 9) resulted in moderate tumor growth inhibition compared to the control; (**B**) For PSA measurements, blood was taken after four weeks of treatment via the tail vein, and serum PSA levels were determined by Advia CentaurXP Immunoassay System. Values were indicated as ng/mL ± SD; (**C**) Expression of AR and PKARIα was analyzed by Western blotting of tumor lysates obtained after one week of treatment. Protein values were normalized with GAPDH.

We next investigated the *in vivo* effects of single or dual targeting of AR and PKARIα with second generation ODNs using subcutaneously growing tumors in immunodeficient mice. Similar to the treatment regime applied *in vitro*, ODNs were injected intraperitoneally three times in the first week and two times per week for the subsequent three weeks, so that the total treatment time was four weeks. All effects were compared with those of a non-targeting control ODN (ODN_Ctrl). On the whole, treatment was non-toxic to the mice, indicated by the absence of a change in animal behavior or body weight. As illustrated in Figure 3A, single targeting with the ODN_AR reduced the mean tumor volume to 2.70 ± 2.97 cm^3^ (*n* = 9, range: 3.49–0 cm^3^) compared to the control (mean tumor volume = 3.79 ± 2.41 cm^3^, *n* = 9, range: 5.43–0.66 cm^3^). The ODN_PKA alone had a similar reducing effect resulting in a mean tumor volume of 2.74 ± 2.05 cm^3^ after four weeks of treatment (*n* = 10, range: 7.84–0 cm^3^). Statistical analysis revealed no significant differences comparing controls and ODN_AR or ODN_PKA single targeting. However, combined treatment of LNCaP tumors with ODN_AR and ODN_PKA over four weeks resulted in a significant growth inhibition (mean tumor volume = 1.07 ± 1.05 cm^3^, *n* = 12, range: 4.09–0 cm^3^, * *p* = 0.008, Mann Whitney-*U* test) compared to the control. Serum PSA levels were also reduced by treatment with ODNs, although there were no significant differences measured between single and dual targeting (Figure 3B, *p* > 0.05, unpaired *t*-test). In particular, ODN_AR reduced serum PSA levels to 121.4 ± 153.5 ng/mL (*n* = 5, range: 361.4–0.39 ng/mL), ODN_PKA to 92.2 ± 107.4 ng/mL (*n* = 5, range: 224.8–0.16 ng/mL) and combined treatment to 112.7 ± 97.9 ng/mL (*n* = 6, range: 269.8–1.5 ng/mL) compared to 405.7 ± 345.7 ng/mL (*n* = 5, range: 998.9–105.3 ng/mL) in the ODN_Ctrl group.

**Figure 4:**
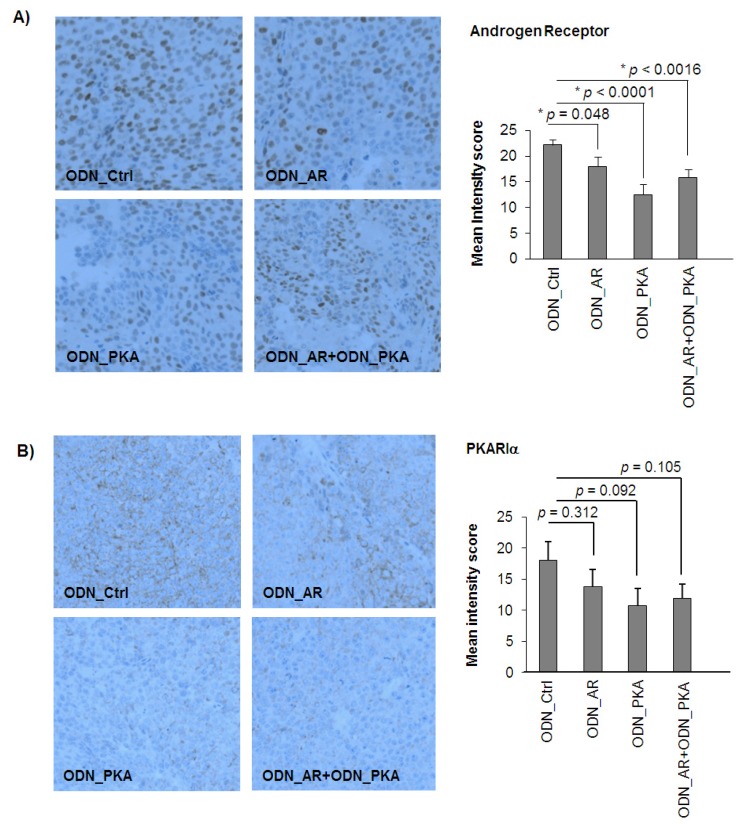
Immunohistochemical detection of AR and PKARIα in LNCaP xenograft tumors. After four weeks of treatment, animals were sacrificed and tumors excised, fixed and embedded in paraffin. A tissue microarray was established, as described in the Material and Methods. Sections (4 μm) were stained for AR (α-AR) (**A**) and PKARIα (α-PKARIα) (**B**) and counterstained with hematoxylin. Images show representative tumor cores (magnification 200×). The AR was mainly found in the nucleus, whereas PKARIα was primarily detected in the cytoplasm. Slides were scored automatically by using TissueQuest software, and intensity scores were expressed as mean values ± S.E.M. (*p < 0.05, *t*-test, two-sided).

We further determined the expression of AR and PKARIα by Western blotting using lysates of LNCaP tumors, which were harvested after one week of treatment. As depicted in Figure 3C, both ODNs were effective in reducing their target proteins when administered alone or in combination. To estimate target protein expression in LNCaP tumors after four weeks of treatment, we performed immunohistochemical staining for AR and PKARIα. As demonstrated in Figure 4A,B, nuclear AR expression was still significantly reduced in tumors treated with ODN_AR over four weeks (* *p* = 0.048), although the effects were less pronounced compared to the Western blot results after the first week of treatment. In tumors treated with ODN_PKA, we measured reduced levels of PKARIα, which was typically detected in the cytoplasm (*p* = 0.092). Similar to the *in vitro* Western blot results, we observed a *vice versa* inhibition of target protein expression after single targeting, in that AR levels were also reduced by the ODN_PKA (*p* = 0.0001), confirming our previous findings with siRNAs [21]. Both AR and PKARIα expression levels were also reduced after combined targeting; however, combined targeting did not enhance downregulation compared to single targeting. Together, these data show that combined targeting of AR and PKARIα has enhanced tumor-inhibitory potential over single treatments in androgen-sensitive LNCaP tumors *in vivo*.

### 2.3. Complete Tumor Remission in Castration-Resistant LNCaPabl Tumors by Combined Targeting of AR and PKARIα

**Figure 5:**
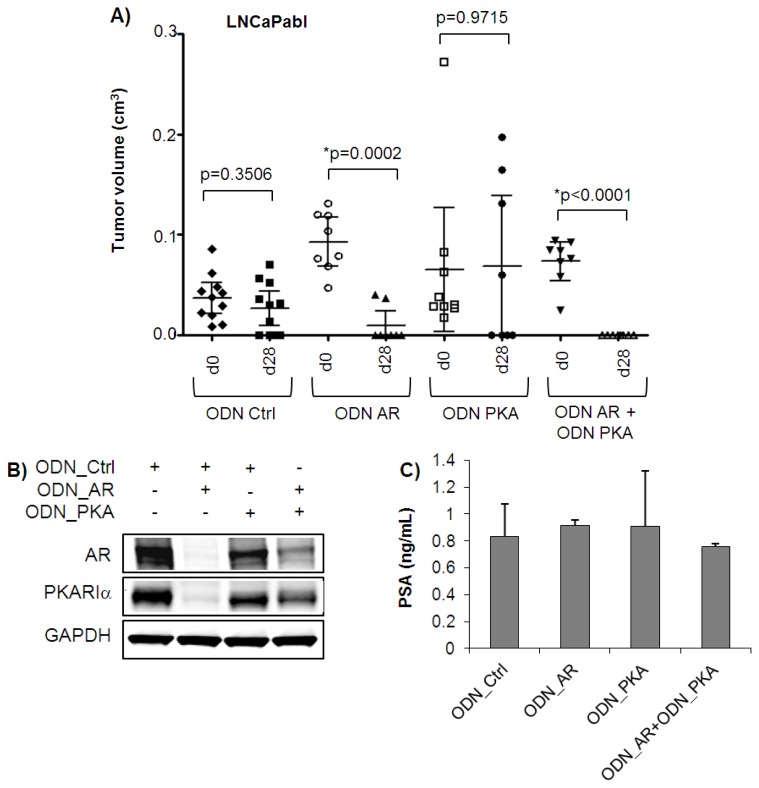
Effects of ODN_AR and ODN_PKA either alone or in combination in LNCaPabl tumors *in vivo*. For establishment of castration-resistant LNCaPabl tumors, mice were castrated one week before tumor cell injection. (**A**) Administration of ODNs was started at day 0 (d0) and tumors were harvested at day 28 (d28). Tumor volumes were determined by caliper measurement and displayed individually. In addition, mean tumor volume ± S.E.M was indicated. Significant reduction of tumor volumes was detected after treatment with ODN_AR alone (*p = 0.0002, *n* = 8), as well as in combination with ODN_PKA (*p < 0.0001, *n* = 8, paired *t*-test). Neither the ODN_Ctrl (*n* = 11) nor the ODN_PKA (*n* = 8) significantly affected tumor growth; (**B**) Expression of AR and PKARIα was determined by Western blotting in tumors harvested after one week of treatment. GAPDH was used as internal control; (**C**) For PSA measurements, blood was taken at the end of treatment, and serum PSA levels were determined by Advia CentaurXP Immunoassay System. Values were indicated as ng/mL ± SD.

For establishment of castration-resistant LNCaPabl tumors, mice were castrated one week before tumor cell injection. ODNs were administered intraperitoneally over four weeks. In general, tumor growth was very heterogeneous. As summarized in Figure 5A, tumor growth was significantly reduced by treatment with the ODN_AR over four weeks (* *p* = 0.0002, paired *t*-test, *n* = 8). Correspondingly, Western blot analysis revealed efficiently reduced AR protein levels and also strongly reduced PKARIα expression after treatment of mice with ODN_AR alone (Figure 5B). Strikingly, combined treatment with ODN_AR and ODN_PKA resulted in complete remission of LNCaPabl tumors (* *p* = 0.036, paired *t*-test, *n* = 8), suggesting an enhanced effect of combination treatment. Intriguingly, however, mean tumor volume was not reduced in mice treated with the ODN_PKA alone (*n* = 8). It should be noted that mean tumor sizes, as well as tumor growth over four weeks was smaller in the LNCaPabl than in the LNCaP model. This is most likely due to castrate conditions, which are thought to strongly influence treatment responses. Influenced by the randomization process, the tumors of the control group were smaller than those of the treatment groups at the beginning of treatment. Comparison of tumors sizes between the start and the end of treatment yielded significant tumor growth inhibition by the ODN_AR alone and by the combination. A small, but not significant, reduction of mean tumor volume was also seen in the group treated with the control ODN. Solely in the ODN_PKA treatment group, some tumors increased in size during the four week treatment period, which resulted in an unchanged mean tumor volume. PSA levels did not correlate with tumor sizes, most likely due to the low levels of serum PSA produced by LNCaPabl tumors (below 1 ng/mL) (Figure 5C) and could therefore not be used as an additional indirect marker for tumor size. Western blot analysis showed moderate reduction of PKARIα protein levels in one tumor harvested after one week of treatment, again indicating heterogeneous treatment response. One may speculate that delivery of the ODN to the tumor site was inefficient in this group of mice or that castration conditions of the LNCaPabl xenografts model may influence bioavailability and tumor uptake of the ODNs. There is also evidence of truncated AR variants lacking the ligand binding domain in castration resistant prostate cancer [36], which would not be targeted by the ODN_AR targeting the hormone binding domain used in our study. Although the impact of AR variants on prostate tumor growth and progression is still unknown, they may contribute to LNCaPabl cell survival.

### 2.4. Effects of Single and Combined Targeting of AR and PKARIα on Apoptosis and Tumor Angiogenesis

**Figure 6:**
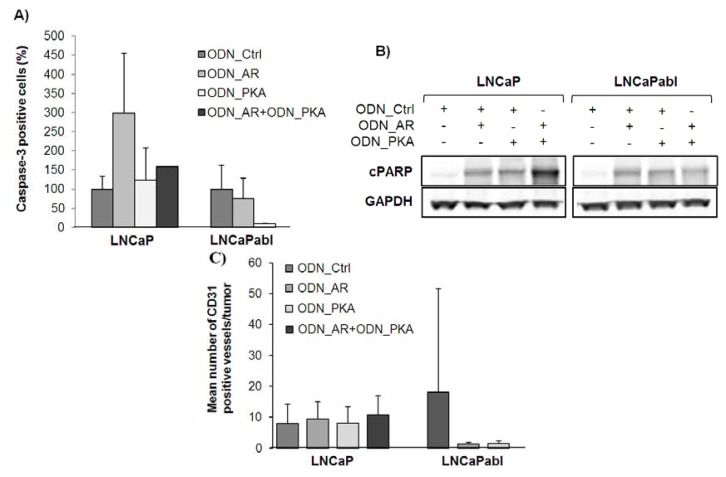
Effects of single or combined treatment with ODN_AR and ODN_PKA on apoptosis and angiogenesis. Athymic nude mice bearing LNCaP and LNCaPabl tumors, respectively, were treated with ODN_AR and ODN_PKA, either alone or in combination, over four weeks. At day 28 of treatment, animals were sacrificed, tumors excised and fixed in formalin and further embedded in paraffin. (**A**) The number of caspase-3 positive cells per tumor was assessed by immunohistochemical staining for cleaved caspase-3 after four weeks of treatment. A tissue microarray was established from LNCaP tumor samples as described under Material and Methods. The number of caspase-3 positive cells was evaluated automatically by using TissueQuest software. In LNCaPabl tumors, the number of caspase-3 positive cells was counted manually; (**B**) Western analysis of lysed LNCaP and LNCaPabl tumor samples, which were harvested after one week of treatment. Immunoblot was performed with an antibody recognizing cPARP p85 fragment. GAPDH was used as loading control; (**C**) Blood vessel count was determined by immunohistochemical staining for CD31 in LNCaP and LNCaPabl tumors harvested after four weeks of treatment. The number of CD31 positive vessels was counted manually and displayed as the mean number of vessels normalized with tumor size ± SD.

We next investigated the effects of combined and single targeting with ODN_AR and ODN_PKA on apoptosis. As summarized in Figure 6A, we detected a strong—though not statistically significant— increase in caspase-3 positive cells in LNCaP tumors treated with ODN_AR over four weeks compared to the ODN_Ctrl (*p* = 0.186), confirming previous data reporting on induction of apoptosis by inhibiting AR expression [16–21]. There was also a minor increase of caspase-3 levels in tumors of the combined treatment group and in those treated only with ODN_PKA (*p* > 0.05). In LNCaPabl tumors, by contrast, neither treatment with ODN_AR nor with ODN_PKA was able to increase the number of caspase-3 positive events. In fact, we measured even lower levels of caspase-3 in the treatment groups compared to the control (*p* > 0.05). It is conceivable that the time point for caspase-3 measurement is too late, because apoptosis may already be exhausted after four weeks of treatment. We therefore determined cPARP levels in tumors harvested after one week of treatment by Western blotting. At this time point, there were still small LNCaPabl tumors found in the combined treatment group. Figure 6B shows that cPARP levels were increased in LNCaP tumors in all treatment groups with the strongest effect found in tumors treated with ODN_AR and ODN_PKA together. Increased cPARP levels were also detected in LNCaPabl tumors, suggesting initial induction of apoptosis, but cessation towards the later phase of treatment.

### 2.5. Effects of Single and Combined Targeting of AR and PKARIα on Tumor Angiogenesis

A correlation between angiogenesis and prostate tumor growth has been shown in different animal models, as well as in human tissue. Kozlowski *et al*., for example, showed a strong correlation between hormonal status and blood supply in animal models of hormone-dependent prostate cancer [37]. In prostate cancer patients, several groups have demonstrated that microvessel density correlates with Gleason score and metastasis [38–40]. Pallares and colleagues even postulated that the “initiation switch” of angiogenesis might be an early event in prostate cancer, since they detected neovessels in lesions of high grade prostatic intraepithelial neoplasia (PIN) and an increase in the number of microvessels in advanced tumors [41]. In a very recent study published by Bates and colleagues, it was demonstrated that the co-inoculation of endothelial cells and prostate luminal or basal epithelial cells in mice significantly improves growth of prostatic tissue as compared with cell lines alone [42]. Recent studies by Godoy *et al*. have shown that the AR, the key regulatory molecule of the androgen signaling cascade, is expressed and functionally active in prostate endothelial cells [43]. In line with that, it was shown that inhibition of the androgen signaling pathway through androgen deprivation or siRNA-mediated AR knockdown not only affects tumor growth, but also microvessel density [44,45].

We therefore analyzed whether single or dual targeting of AR and PKARIα had an impact on tumor angiogenesis. For this purpose, we determined the number of CD31 positive blood vessels by immunohistochemical staining of tumors, which were harvested after four weeks of treatment. Neither single agents nor a combination of the ODNs affected the number of CD31 positive vessels in LNCaP tumors (*p* > 0.05, Figure 6C). However, the ODN_AR dramatically—(though not significantly, due to the limited number of tumors eligible for analysis, *p* > 0.05)—reduced the number of CD31 positive vessels compared to the ODN_Ctrl in castration-resistant LNCaPabl tumors, indicating a deleterious effect on angiogenesis. Again, it should be considered that castration may strongly influence treatment conditions in the LNCaPabl model. Interestingly, a reduced blood vessel count was also observed in ODN_PKA treated tumors (*p* > 0.05), although there was no tumor growth-inhibitory effect seen with the ODN_PKA. Since LNCaPabl tumors in general were very small, one could speculate that tumor supply with nutrients and oxygen was still possible through diffusion and compensated the loss of blood vessels. Overall, we observed that untreated LNCaPabl tumors had about twice the number of vessels compared to LNCaP tumors, a finding that has also been described previously by Gustavsson *et al*. in a similar androgen-independent LNCaP subline (LNCaP-19) [46].

## 3. Experimental Section

### 3.1. Drugs and Chemicals

Long half-life second generation oligonucleotides (ODNs) with 2′-*O*-(2-methoxy)ethyl modifications at the 5′ and 3′ ends, respectively, and phosphorothioated internucleotide linkages were purchased from GenXpress. ODNs were designed against the ligand binding domain of the AR (ODN_AR 5′-*u*g*c*ugaagagtagc*a*g*u*g-3′) and the RIα subunit of PKA (ODN_PKA 5′-*g*c*g*ugcctcctcac *t*g*g*c-3′). A negative unspecific control was used to exclude unspecific side effects (ODN_Ctrl 5′-*a*g*a*ggcttgcacag*t*g*c*a-3′). Modified bases are indicated by an asterisk. To evaluate transfection efficiency, cells were transfected with a fluorescently-labeled single-stranded control RNA-ODN (cell signaling) for 24 h. Fluorescence was detected by a Becton-Dickinson FACSCalibur cytometer with CellQUEST Pro Software (Becton-Dickinson, San Jose, CA, USA). The synthetic androgen methyltrienolone (R1881) was purchased from Perkin Elmer, Waltham, MA, USA.

### 3.2. Cell Lines

LNCaP prostate cancer cells were obtained from the American Type Culture Collection, USA. LNCaP cells were cultured as described previously [21]. LNCaPabl cells have previously been generated from LNCaP by long-term androgen ablation, thereby gaining the characteristics of CRPC, including increased AR protein levels, hypersensitivity to low levels of androgens and the ability to grow in castrated mice [47,48]. LNCaPabl cells were cultured in RPMI 1640 with 10% charcoal-stripped FCS (CS-FCS), 1% Glutamax and antibiotics. All cells were maintained at 37 °C in a humidified atmosphere of 5% CO2. The identity of the used cell lines was confirmed by short tandem repeat analysis.

### 3.3. *In Vitro* Transfections with the ODNs

Semi-confluent cells were transfected with the ODNs using oligofectamine (Invitrogen, Vienna, Austria), following the manufacturer’s protocol. Transfection was performed in antibiotic-free medium supplemented with 5% FCS on polylysine coated plates (0.03 mg/mL, Sigma, Vienna, Austria). Cells were either transfected with the ODN_AR (250 nM) and the ODN_PKA (250 nM) for dual targeting or with 250 nM of ODN_AR or ODN_PKA together with 250 nM ODN_Ctrl for single treatments. All effects were compared with those of a control group treated with 500 nM ODN_Ctrl. Transfections were done on day 1, 3 and 5 in the first week. In weeks 2–4, treatment was performed two times per week. LNCaP cells were treated with the ODNs in medium supplemented with 0.1 nM R1881, LNCaPabl cells in charcoal-stripped medium without androgen. Cells were harvested with trypsin and counted with a Casy cell counter (Schärfe System, Reutlingen, Germany) weekly. Equal cell numbers were re-plated, and the cell number was followed cumulatively.

### 3.4. Flow Cytometric Detection of Apoptotic Cells

Cells were harvested by trypsinization at day 28 of treatment, washed with PBS and centrifuged for 5 min at 1200 rpm. The resulting cell pellet was re-suspended in 300 μL Nicoletti solution (50 μg/mL propidium-iodide, 0.1% sodium-citrate, 0.1% Triton-X in aqua Bidest.) and incubated for 24 h at 4 °C. Fluorescence was detected by a Becton-Dickinson FACSCalibur cytometer with CellQUEST Pro Software (Becton-Dickinson, San Jose, CA, USA). The percentage of apoptotic cells was calculated from the subG1 phase population in a logarithmic scaled *x*-axis histogram using the FL2-H channel. Analysis of cell cycle distribution was carried out using the same data sets in a linear scaled *x*-axis histogram using the FL2-A channel (DDM-function of the software, Becton, Dickinson).

### 3.5. Immunoblotting

Western blot analysis was performed as described previously [21]. Whole cell extracts or small tumor pieces, which were shock-frozen after harvesting, were re-suspended in lysis buffer [20 mM NaH_2_PO_4_, 1 mM EDTA, 10% glycerol, 0.1 nM PMSF, 0.5 nM NaF, 0.5% Protease Inhibitor Cocktail Set III (Calbiochem, Germany), 0.5% Phosphatase Inhibitor Cocktail 2 (Sigma, Vienna Austria)] and shaken for 1 h at 4 °C. After lysis of the cells, the supernatant was collected by centrifugation at 10,000 rpm for 10 min. Protein content was determined by the Bradford assay. Equal amounts of protein (100 μg) were loaded and resolved in 4%–12% Bis-Tris gels (Invitrogen, Carlsbad, CA, USA) and subsequently transferred onto nitrocellulose membranes (Invitrogen, Carlsbad, CA, USA). Membranes were blocked for 1 h by using Starting Block buffer (THP Medical Products, Vienna, Austria), followed by overnight incubation with primary antibodies. The primary antibodies used for immunoblotting were as follows: AR (1:200, Biogenex, San Ramon, CA, USA), PKARIα (1:500, BD Transduction Laboratories, Franklin Lakes, USA), cPARP p85 fragment (1:1000, Promega, Madison, WI, USA), Mcl-1 (1:500, Santa Cruz, Dallas, TX, USA) and GAPDH (1:100,000, Millipore, Temecula, CA, USA). After 4 times 5-min of washing with TBS containing 0.05% Tween 20 (TBST), membranes were incubated for 1 h with fluorescence-labeled secondary antibodies (Molecular Probes, Oregon, OR, USA) and then washed again with TBST, as previously described. The membranes were finally scanned and quantified using the Odyssey infrared imaging system (LiCor Biosciences, Lincoln, The Netherlands).

### 3.6. Establishment and Treatment of Human Prostate Tumor Xenografts in Nude Mice

Animal protocols were approved by the Austrian Federal Ministry for Education, Science and Culture (BMWF-66.011/0130-II/10b/2009, BMWF-66.011/0116-II/3b/2011). All efforts were made to minimize suffering of the animals. We used male nude mice (BALB/c/*nu*/*nu*, 4–6 weeks old), which were purchased from Charles River Laboratories (Sulzfeld, Germany) and housed under pathogen-free conditions. Xenografted tumors were grown by subcutaneous implantation of a 0.1 mL suspension of 2 × 10^6^ LNCaP cells mixed with 0.1 mL matrigel (BD Biosciences, Bedford, MA, USA) into both the right and left flanks of mice, respectively. LNCaPabl cells (2 × 10^6^ cells mixed with matrigel in a 1:1 ratio) were injected into castrated mice. Castration was performed by orchiectomy of anesthetized animals one week before tumor cell injection. Mice were initially anesthetized by inhalation with 3%–4% methoxyflurane. For maintenance of anesthesia, the concentration of methoxyflurane was reduced to 1.25%–1.75%. When the tumors became palpable, mice were randomly divided into four treatment groups: ODN_Ctrl (10 mg/kg body weight), ODN_Ctrl (5 mg/kg body weight) + ODN_AR (5 mg/kg body weight), ODN_Ctrl (5 mg/kg body weight) + PKA_AR (5 mg/kg body weight), ODN_AR (5 mg/kg body weight) + ODN_PKA (5 mg/kg body weight). ODNs were dissolved in sodium chloride solution and administered intraperitoneally (*i.p.*) at a final concentration of 10 mg/kg mouse three times in the first week and twice from week 2 to 4. After one week of treatment, one mouse per group was sacrificed and the tumors harvested for further evaluations. The remaining animals were treated for another three weeks. Two days after the last injection, all mice were killed. Tumor sizes were determined by caliper measurements and calculated with the formula length × width × height × 0.5236. Each tumor was measured individually. For PSA measurements, blood was taken via the tail vein. Tumors were subdivided into two pieces and either frozen in liquid nitrogen for Western blot analysis or fixed in buffered formalin (4.5%) and embedded in paraffin for further immunohistochemical staining.

### 3.7. PSA Measurement

PSA values were determined in mouse serum by Advia CentaurXP Immunoassay System (Siemens, Munich, Germany).

### 3.8. Construction of LNCaP Tissue Microarray (TMA)

A TMA was created from LNCaP tumors using a manual tissue microarrayer from paraffin-embedded tumor tissue. The LNCaP TMA contained 60 tissue cores (core diameter 1.0 mm) from tumors, which were harvested after 1 week (3 cores of each tumor, 2 tumors per treatment group), 228 cores from tumors, which were harvested after 4 weeks of treatment (3 cores per tumor, 4–12 tumors per treatment group) and 18 cores from cell culture controls (3 cores per treatment). For cell culture controls, LNCaP cells were transfected as described above. After 3 days of treatment, cells were harvested with trypsin, washed with PBS and counted. Cells (4–8 × 10^6^) were then suspended with 450 μL citrate-plasma and 11.3 μL 1 M calcium chloride. Then, 45 μL thrombin (120 NIH-U/mg protein, Sigma (Vienna, Austria), T4648-1KU) were added and the solution stirred until coagulation. The resulting pellet was then put into a histosette and fixed with formaldehyde for 2 h. Afterwards, the pellet was dehydrated and fixed in tissue-Tek VIP (Sakura, Torrance, CA, USA) overnight and further embedded in paraffin.

### 3.9. Immunohistochemistry

Immunohistochemical staining was done on formalin-fixed and paraffin embedded 4-μm sections using the Ventana autostainer model Discover XT (Ventana Medical System, Roche) with an enzyme-labeled biotin streptavidin system and solvent-resistant 3,3′-diaminobenzidine Map kit. Slides were pretreated with Tris borate EDTA buffer (pH 7.8, Roche) for 48 min. The following antibodies were used: AR (rabbit polyclonal, Eubio), PKARIα (rabbit polyclonal, AbTDSerotec), CD31 (rabbit polyclonal, Abcam, Cambridge, UK), cleaved caspase-3 (rabbit polyclonal, Cell Signaling). Specificity of staining was controlled by including an unspecific control antibody (DAKO, Denmark). Slides were counterstained with hematoxylin (Roche). LNCaP TMA samples were scored automatically by using TissueQuest software (TissueGnostics, Vienna, Austria). CD31 stainings were scored manually by two independent researchers. In LNCaPabl, CD31 and caspase-3 expression were evaluated manually.

### 3.10. Statistics

Gaussian distribution was calculated by the Kolmogorov-Smirnov test and the non-parametric Kruskal-Wallis test. The Mann Whitney-*U* test or parametric tests (two-way ANOVA, unpaired two-sided Student’s *t*-test) were used to assess statistical significances, unless otherwise indicated. Statistical calculations were performed with SPSS software. * *p* < 0.05 was considered as statistically significant. Data were expressed as means with standard deviations (SD) or standard error of mean (SEM).

## 4. Conclusions

AR and PKARIα are implicated in the activation of AR signaling and progression of prostate cancer towards castration resistance. While there is evidence that single targeting of either AR [16–21] or PKARIα [29,49] has tumor-inhibitory potential, the data presented in this study are the first showing the effects of combined targeting of these two molecules in prostate cancer *in vivo*. In summary, our results show that combined treatment of prostate tumors with antisense oligonucleotides targeting AR and PKARIα is more effective than single treatments in androgen-sensitive LNCaP and castration-resistant LNCaPabl tumors. Our findings confirm previous data by Hensley and colleagues, who showed that the efficacy of the PKARIα antisense ODN GEM231 is enhanced by androgen ablation treatment in the LNCaP model [49]. Further studies are warranted to validate the effects of combined inhibition of AR and PKARIα in other prostate cancer tumor models.

## Abbreviations

AR: androgen receptor
CRPC: castration-resistant prostate cancer
PKARIα: regulatory subunit type I alpha of protein kinase A
PSA: prostate-specific antigen
ODN: oligodeoxynucleotide
TMA: tissue microarray
MVD: microvessel density
PARP-1: poly(ADP-ribose)-polymerase 1
